# Deep Sea Water Improves Abnormalities in Lipid Metabolism through Lipolysis and Fatty Acid Oxidation in High-Fat Diet-Induced Obese Rats

**DOI:** 10.3390/md15120386

**Published:** 2017-12-11

**Authors:** Wei-Tang Chang, Tsung-Yueh Lu, Ming-Ching Cheng, Hsun-Chi Lu, Mei-Fang Wu, Chin-Lin Hsu

**Affiliations:** 1 School of Nutrition, Chung Shan Medical University, Taichung 404, Taiwan; lko78779@hotmail.com (W.-T.C.); liop520@yahoo.com.tw (H.-C.L.); 2 Department of Industrial Engineering and Systems Management, Feng Chia University, Taichung 404, Taiwan; cs320866@yahoo.com.tw (T.-Y.L.); mfwu@fcu.edu.tw (M.-F.W.); 3 Department of Health Food, Chung Chou University of Science and Technology, Changhua 510, Taiwan; m25522@yahoo.com.tw; 4 Department of Nutrition, Chung Shan Medical University Hospital, Taichung 404, Taiwan

**Keywords:** deep sea water, high fat-diet, Wistar rat, gene expression

## Abstract

Deep sea water (DSW) is a natural marine resource that has been utilized for food, agriculture, cosmetics, and medicine. The aim of this study was to investigate whether DSW has beneficial lipid metabolic effects in an animal model. Our previous in vitro study indicated that DSW significantly decreased the intracellular triglyceride and glycerol-3-phosphate dehydrogenase activity in 3T3-L1 adipocytes. DSW also inhibited the gene levels of adipocyte differentiation, lipogenesis, and adipocytokines, and up-regulated gene levels of lipolysis and fatty acid oxidation. In the present study, the results showed that body weight, liver, adipose tissue, hepatic triglycerides and cholesterol, and serum parameters in the high-fat diet (HFD) + DSW groups were significantly lower compared to the HFD group. Moreover, the fecal output of total lipids, triglycerides, and cholesterol in the HFD + DSW groups was significantly higher than that of the HFD group. Regarding gene expression, DSW significantly increased the gene levels of lipolysis and fatty acid oxidation, and decreased the gene levels of adipocytokine in the adipose tissue of rats with HFD-induced obesity. These results indicate a potential molecular mechanism by which DSW can suppress obesity in rats with HFD-induced obesity through lipolysis and fatty acid oxidation.

## 1. Introduction

Obesity is one of the most pervasive public health problems in developed and developing countries. Obesity plays a critical role in the causal path towards the development of metabolic syndrome, and is a compilation of risk factors that predispose individuals to the development of cardiovascular diseases, type 2 diabetes, hypertension, and certain cancers [1,2,3]. Elevated mortality rates have also been related to overweight- and obesity-related health problems in adults [4].

Some weight loss drugs have serious side effects [5]. For instance, sibutramine, widely used after approval by the U.S. FDA in 1997, was withdrawn from the U.S. market in October 2010 due to concerns about the increased cardiovascular events and strokes, while rimonabant was withdrawn from the European market in 2009 due to the unacceptable psychiatric side effects (increased risk of suicide). Many natural compounds have been used to treat obesity, including polyunsaturated fatty acids (PUFAs), monounsaturated fatty acids (MUFAs), conjugated linoleic acid (CLA), phenolic compounds, soybean, plant sterols, dietary calcium, and dietary fiber [6]. Beyond this, some studies have demonstrated that deep sea water (DSW) acts as a natural anti-obesity agent by inhibiting adipogenesis in 3T3-L1 adipocytes and ob/ob mice [7,8]. DSW is a functional water resource that has applications in food, agriculture, cosmetics, and other areas in many countries such as Taiwan, Japan, and South Korea. Dietary DSW offers many health benefits because it is enriched with minerals, such as magnesium (Mg^2+^), calcium (Ca^2+^), and potassium (K^+^) [9,10]. Many studies have indicated that DSW enriched with Mg and Ca prevents and treats several chronic diseases, including diabetes and obesity [11,12]. Our previous study indicated that drinking deep sea water inhibits the adipogenesis of 3T3-L1 adipocytes and attenuates high-fat/cholesterol diet-induced cardiovascular disorders in hamsters [13,14]. However, the literature indicates that the mechanism underlying the lipid metabolic effects of DSW in rats with high-fat diet-induced obesity remain unclear.

The objective of this study was to investigate the lipid metabolic effect of DSW on rats with high-fat diet (HFD)-induced obesity. Our study also aimed to confirm whether the metabolic effect of DSW is caused by regulating lipogenesis, lipolysis, or fatty acid oxidation molecular pathways.

## 2. Results

### 2.1. Effect of DSW on Body Weight, Water Intake, Food Intake, Energy Intake, Organ Size, and Adipose Tissue in Rats with HFD-Induced Obesity

As shown in Table 1, after six weeks of feeding, final body weight and perirenal fat were significantly lower in the HFD + DSW (1–5×) groups (*p* < 0.05) compared to the HFD group. The weight of epididymal fat of the HFD + DSW (3× and 5×) was significantly lower (*p* < 0.05) than that of the HFD group. The weight of the liver in several HFD + DSW groups (2×, 3×, and 5×) was significantly lower (*p* < 0.05) compared to the HFD group. The water intake of the HFD + DSW (3× and 5×) groups was significantly higher (*p* < 0.05) than the water intake of the HFD group. There were no significant differences in food intake, energy intake, and organ sizes of the heart, spleen, lungs, and kidneys among the five groups.

### 2.2. Effect of DSW on the Serum Biochemical Indicators in Rats with HFD-Induced Obesity

As shown in Table 2, the serum glucose level of the HFD + DSW (2–5×) groups was significantly lower (*p* < 0.05) than that of the HFD group. The serum levels of triglycerides, ALT, and insulin in the HFD + DSW (1–5×) groups were significantly lower (*p* < 0.05) than those in the HFD group. The serum free fatty acids levels of of the HFD + DSW (1×, 3×, and 5×) groups were significantly lower (*p* < 0.05) than that of the HFD group. The total serum cholesterol levels of the HFD + DSW (1×, 2×, and 5×) groups were significantly lower (*p* < 0.05) than that of the HFD group, whereas the level of serum K^+^ in the HFD + DSW (5×) group was significantly higher (*p* < 0.05) compared to the HFD group. Increased serum Mg^2+^ levels were also observed in the HFD groups supplemented with DSW (2×, 3×, and 5×). There were no significant differences in the serum levels of LDL cholesterol, uric acid, creatinine, Na^+^, and Cl^−^ among the five groups (*p* > 0.05).

### 2.3. Effect of DSW on Hepatic Antioxidant Enzyme and Total Hepatic and Fecal Lipids, Triglycerides, Cholesterol, and Mg^2+^ in Rats with HFD-Induced Obesity

**Figure 1:**
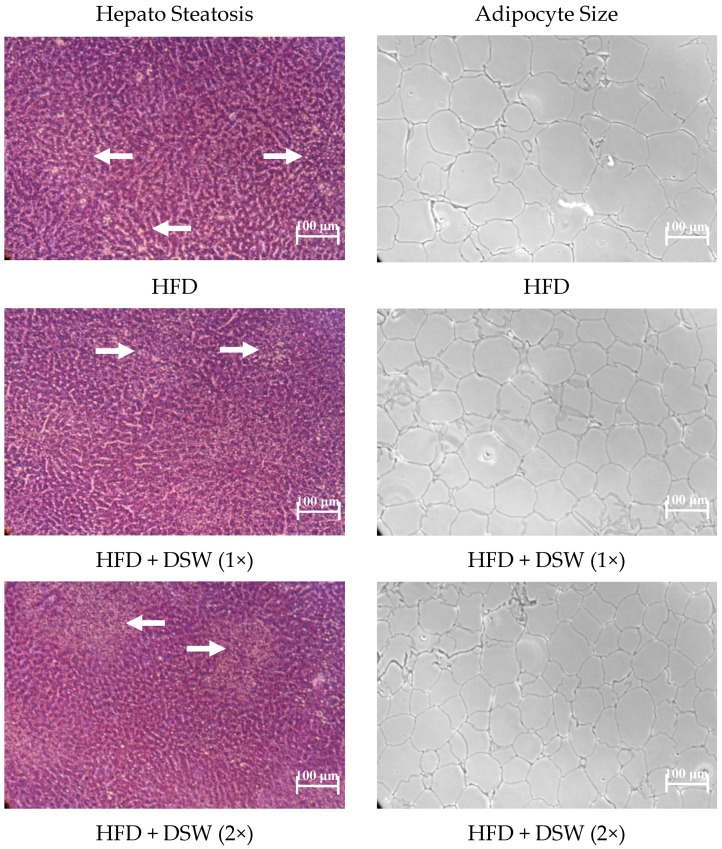

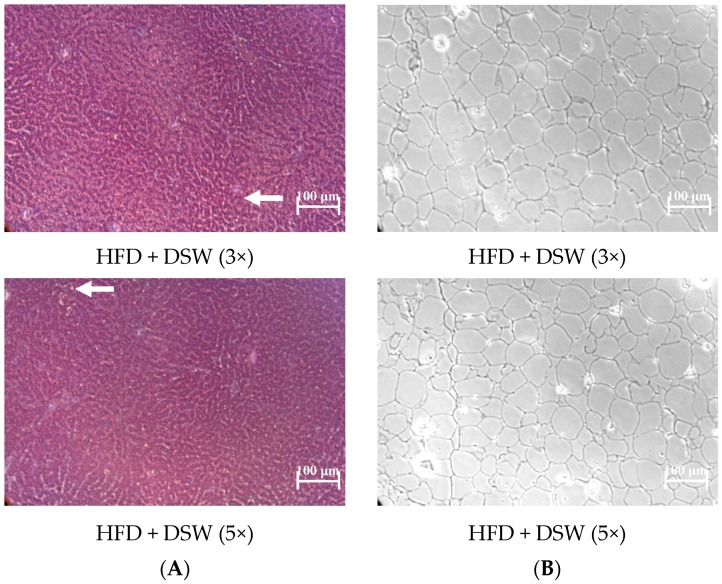
Effect of deep sea water on hepatosteatosis (**A**) and adipocyte size (**B**) of rats with obesity induced by a high-fat diet. The liver tissue and adipocytes were stained with hematoxylin and eosin (H&E). Original magnification: 200×.

As shown in Figure 1A, hematoxylin and eosin staining (H&E staining) showed macrovesicular fat accumulation in the HFD group. The HFD + DSW (1–5×) groups showed microvesicular fat accumulation. The sizes of the adipocytes in the HFD + DSW (1–5×) groups were smaller than those in the HFD group (Figure 1B). The effects of DSW on hepatic antioxidant enzyme and the total hepatic and fecal lipids, triglycerides, cholesterol, and Mg^2+^ in rats with HFD-induced obesity are shown in Table 3. There were no significant differences in the hepatic TEAC and glutathione S-transferase (GST) levels between the HFD and the HFD + DSW (1–5×) groups (*p* > 0.05). The hepatic thiobarbituric acid reactive substances (TBARS) values in the HFD + DSW (1× and 3×) groups were significantly lower (*p* < 0.05) than those in the HFD group. Hepatic glutathione reductase (GRd) activity in the HFD + DSW (3× and 5×) groups was significantly higher (*p* < 0.05) than the activity in the HFD group. Hepatic total lipids, triglycerides, and cholesterol levels in the HFD + DSW (3× and 5×) groups were significantly lower (*p* < 0.05) than those in the HFD group. Fecal total lipids, triglycerides, and cholesterol levels in HFD+DSW (2–5×) groups were significantly higher (*p* < 0.05) than those in the HFD group. The hepatic Mg^2+^ levels in the HFD + DSW (5×) group were significantly higher (*p* < 0.05) compared to the HFD group.

### 2.4. Effects of DSW on Gene Expressions in Rats with HFD-Induced Obesity

**Figure 2:**
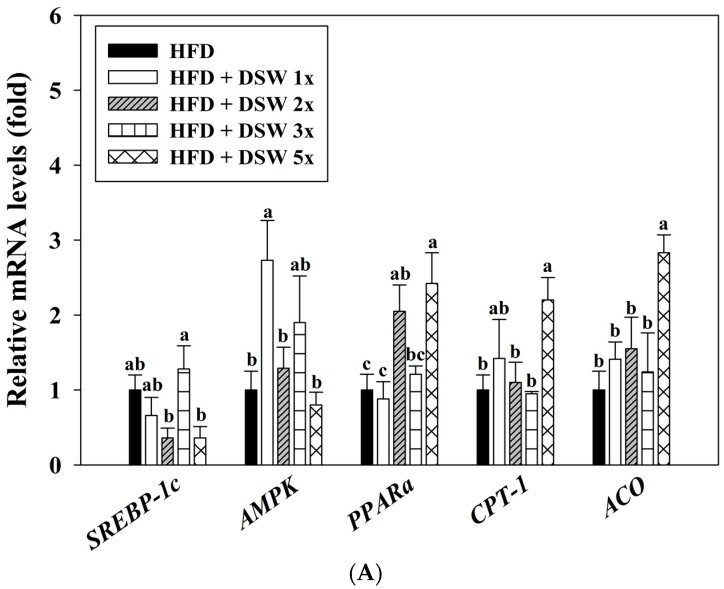

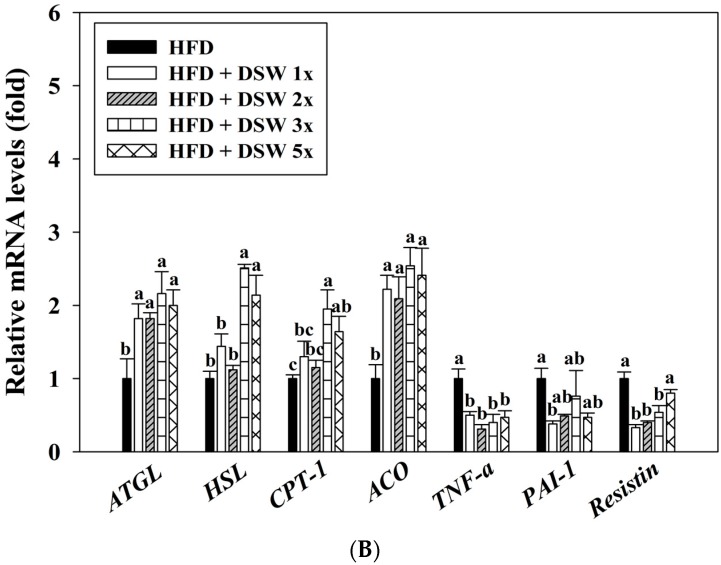
Effect of deep sea water on gene expressions of adipogenesis, lipolysis, fatty acid oxidation, and adipocytokinesin liver (**A**) and adipose tissues (**B**) of rats with obesity induced by a high-fat diet. The reported values are the mean ± SEM (*n* = 8). Mean values with different superscript letters are significantly different (*p* < 0.05). *ACO*, acyl-CoA oxidase; *AMPK*, AMP-activated protein kinase; *ATGL*, adipose triglyceride lipase; DSW, deep sea water; HFD, high-fat diet; *HSL*, hormone-sensitive lipase; *PAI-1*, plasminogen activator inhibitor; *PPARα*, peroxisome proliferator-activated receptor *α*; *SREBP-1c*, sterol regulatory element binding protein; *CPT-1*, carnitine palmitoyl transferase; *TNF-α*, tumor necrosis factor *α*.

As shown in Figure 2, in liver tissue, the gene expression levels of *AMPK* (1×), *PPARα* (2× and 5×), *CTP-1* (5×), and *ACO* (5×) in the HFD + DSW groups were significantly higher (*p* < 0.05) compared to those in the HFD group. In adipose tissue, the levels of *ATGL* and *ACO* (1–5×) *HSL* and *CTP-1* (3× and 5×) in the HFD + DSW groups were significantly higher (*p* < 0.05) than those of the HFD group. The gene expression levels of *TNF-α* (1–5×), *PAI-1* (1×), and *resistin* (1×, 2×, and 3×) in the HFD + DSW groups were significantly lower (*p* < 0.05) than those of the HFD group.

## 3. Discussion

The 3T3-L1 cell line is widely used as a model of adipocyte differentiation and adipose biology. Our previously published data indicated that DSW inhibited intracellular triglycerides and glycerol-3-phosphate dehydrogenase (GPDH) activity in the 3T3-L1 adipocytes [13]. Hwang et al. [7] indicated that the inhibition of intracellular triglycerides in 3T3-L1 adipocytes occurred in a dose-dependent manner when cells were exposed to DSW with a hardness between 0 and 1000 ppm. Watanabe et al. [15] indicated that the cytosolic enzyme GPDH plays an important role in the conversion of glycerol to triglyceride. Our previous studies indicated that some phytochemicals (such as *o*-coumaric acid, rutin, capsaicin, garcinol, and pterostilbene) significantly decrease the amount of intracellular triglycerides and GPDH activity in 3T3-L1 adipocytes [16,17,18].

In in vivo models, obesity is successfully induced in animals (such as mice and rats) by feeding them a high-energy/high-fat diet, including lard, beef tallow, coconut oil, corn oil, soybean oil, or shortening [19]. The rats fed an HFD develop obesity, dyslipidemia, hepatosteatosis, oxidative stress, hyperinsulinemia, and insulin resistance [20,21]. In the present study, we found that feeding obese rats with DSW for six weeks suppressed the increase in body weight, liver size, perirenal fat, and epididymal fat (*p* < 0.05) (Table 1). Hwang et al. [8] indicated that dietary DSW can ameliorate obesity and diabetes in ob/ob mice. Hyperlipidemia is known to increase cardiovascular risk factors, including coronary heart disease, fatty liver disease, hypertension, and carcinogenesis. Lavie and Milani [22] indicated that basal levels of plasma lipids (e.g., a high level of triglycerides and a low level of HDL-cholesterol) are known to have a strong positive correlation with obesity. Our previous data indicated that drinking DSW decreased serum levels of triglycerides, total cholesterol, and TBARS in hamsters fed a high-fat/high-cholesterol diet [14]. We found that consuming DSW for six weeks suppressed the increases in serum levels of glucose, triglycerides, total cholesterol, ALT, free fatty acid, and insulin in obese rats fed an HFD (*p* < 0.05) (Table 2). We also found that serum Mg^2+^ levels in the HFD + DSW groups were significantly higher (*p* < 0.05) than those in the HFD group. Ouchi et al. [23] indicated that adding magnesium sulphate (MgSO_4_) to the diet decreased serum cholesterol and increased serum HDL cholesterol levels in rabbits. Our data indicated that consuming DSW for six weeks decreases hepatic TBARS, total lipids, triglycerides, and cholesterol values in obese rats fed an HFD (*p* < 0.05) (Table 3). In the histology study (H&E staining), the number of hepatic lipid droplets and adipocyte size in the HFD + DSW groups were significantly lower (*p* < 0.05) compared to the HFD group (Figure 1). Our data also indicated that fecal levels of total lipids, triglycerides, and cholesterol in the HFD+DSW groups were significantly lower (*p* < 0.05) than those in the HFD group (Table 3). Our previous study indicated that consuming Ca^2+^ and Mg^2+^-rich DSW increases the fecal output of cholesterol and triglycerides in mice fed a high-cholesterol diet [24].

The modulatory action of DSW in inhibiting obesity in rats fed an HFD is shown in Figure 2. We found that the HFD + DSW groups had significantly higher (*p* < 0.05) levels of lipolysis and fatty acid oxidation-related genes (*AMPK*, *PPARα*, *CPT-1*, and *ACO*) in their liver tissue (Figure 2A). Our data also indicated that the HFD + DSW groups had significantly higher (*p* < 0.05) levels of lipolysis and fatty acid oxidation-related genes (*ATGL*, *HSL*, *CPT-1*, and *ACO*) and lower levels of inflammatory-related ones (*TNF-α*, *PAI-1*, and *resistin*) in their adipose tissue (*p* < 0.05) (Figure 2B). However, this result suggests that DSW can activate lipolysis and the oxidative pathways. Tilg et al. [25] indicated that leptin, resistin, *PAI-1*, and *TNF-α* are the adipocytokines thought to provide a key link between obesity and the related inflammatory response. Ha et al. [26] indicated that supplements with balanced deep-sea water (BDSW) decreased the body weight gain and the adipocytes size, and improved severe liver steatosis in HFD-induced mice. Supplementation with BDSW downregulated the expression of adipogenic, lipogenic, lipolytic, and pro-inflammatory cytokine genes and upregulated the expression of adipokines and β-oxidation genes in the adipose tissue of HFD mice. However, BDSW can be developed as a potential marine drug for the prevention of obesity. Nani et al. [27] indicated that DSW is abundant in minerals, particularly Mg^2+^ and Ca^2+^, when compared to the common mineral water. Those minerals take part in many important physiological processes, such as enzymes activity and energy metabolism. Some studies have reported that DSW may help to reduce lifestyle-associated diseases, such as cardiovascular disease, hyperlipidemia, diabetes, obesity, hypertension, and cancer. Moreover, Bertinato et al. [28] reported that a poor Mg^2+^ status can impair the growth of lean body mass and femoral size (width, weight, and volume), and increase the mass of mesenteric adipose tissue in rats fed an HFD. Recently, it has been found that increasing the intake of dietary Ca^2+^ and Mg^2+^ could enhance the total plant sterol levels and the LDL-cholesterol lowering effect, preventing a diet-induced increase in body weight in this atherogenic diet. There is also evidence that an increased intake of Ca^2+^ and Mg^2+^ may prevent and decrease obesity [29].

**Figure 3:**
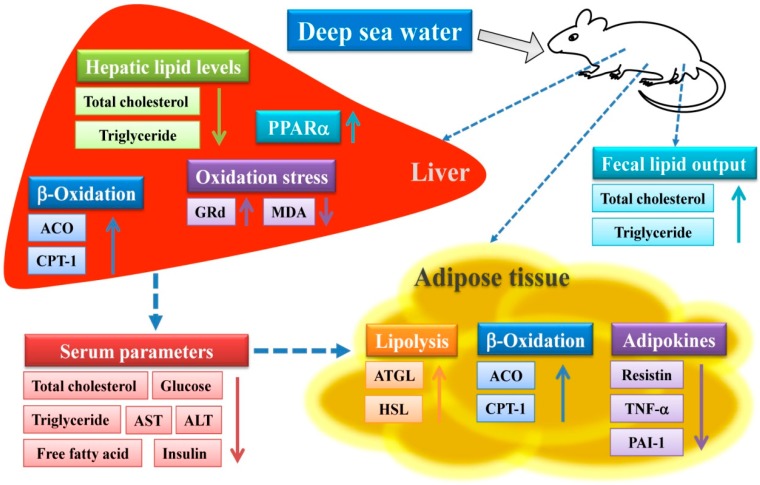
Schematic representation of the mechanism of action through which deep sea water affects obesity induced by a high-fat diet in male Wistar rats. *ACO*, acyl-CoA oxidase; ALT, aspartate aminotransferase; AST, alanine transaminase; *ATGL*, adipose triglyceride lipase; *CPT-1*, carnitine palmitoyl transferase-1; GRd, glutathione reductase; *HSL*, hormone-sensitive lipase; MDA, malondialdehyde; *PAI-1*, plasminogen activator inhibitor-1; *PPARα*, peroxisome proliferator-activated receptor alpha; *TNF-α*, tumor necrosis factor-alpha.

In conclusion, the present results showed that DSW exhibits anti-obesity effects by regulating lipolysis, fatty acid oxidation, and inflammation in rats fed an HFD. These results indicate that lipolysis and fatty acid oxidation are potential molecular mechanisms through which DSW exhibits a beneficial anti-obesity effect in rats with HFD-induced obesity. In Figure 3, we propose a schematic representation of the mechanism of action through which DSW impacts obesity induced by an HFD in male Wistar rats.

## 4. Materials and Methods

### 4.1. Collection of Deep Sea Water (DSW)

Original DSW samples were collected from a depth of approximately 662 m in Hualien Bay in Hualien City, Taiwan. A sufficient supply of original DSW was generously offered by the Taiwan Yes Deep Ocean Water Co., Ltd. from Hualien City, Taiwan. The DSW contained the following major minerals: Mg^2+^ (9600 mg/dL), K^+^ (1000 mg/dL), Na^+^ (900 mg/dL), and Ca^2+^ (4 mg/dL).

### 4.2. Animals, Diets, and Experimental Design

Eight-week-old male Wistar rats were purchased from the National Science Council Animal Center in Taipei, Taiwan. The animals were housed individually in stainless steel cages in an air-conditioned room at 23 ± 2 °C with 55–60% relative humidity, kept on a 12 h light/dark cycle, and given a laboratory rodent chow diet for one week. The rats were fed a high fat-diet (HFD) containing 20% lard. The HFD groups (*n* = 8) were then divided into five groups based on the type of supplemental deep sea water (DSW), given for six weeks: the HFD group (an HFD group with no DSW), HFD + DSW (1×), HFD + DSW (2×), HFD + DSW (3×), and HFD + DSW (5×) groups received the HFD supplemented with DSW of varying hardness (with Mg^2+^ as the major compound).The five groups received DSW with 0, 40, 80, 120, and 200 mg Mg^2+^/kg body weight, respectively (orally administered, mixed with chow diet for rodents). The rats were provided with semi-synthetic diets and water ad libitum throughout the experimental period. The diets were stored in a cold chamber at 4 °C. The rats’ body weight, food intake, and food efficiency were measured every day for six weeks. Food efficiency (g/kcal) was calculated by dividing body weight gain (g/day) by energy intake (kcal/day) over the diet period. After an overnight fast, blood was drawn from the abdominal aorta under carbon dioxide anesthesia, and serum was harvested. The visceral tissues were immediately excised, rinsed, weighed, and frozen in liquid nitrogen. All experimental procedures involving animals were conducted in accordance with the guidelines of the National Institutes of Health (NIH). This experiment was approved by the Institutional Animal Care and Use Committee (IACUC) of Chung Shan Medical University (IACUC Approval No.: 448) in Taichung, Taiwan.

### 4.3. Measurement of Serum Parameters

Blood was placed into a sterile Vacutainer plastic tube (BD Vacutainer, Plymouth, UK). Serum was separated by centrifugation (5000× *g*, 10 min) and transferred to eppendorf tubes. The serum concentrations of triglycerides, glucose, total cholesterol, low-density lipoprotein (LDL) cholesterol, high-density lipoprotein (HDL) cholesterol, aspartate aminotransferase (AST), alanine aminotransferase (ALT), uric acid, creatinine, Mg^2+^, Na^+^, K^+^, and Cl^−^ were measured with commercial kits (Bayer Corporation, Tarrytown, NY, USA). The concentrations of ketone bodies and free fatty acids were measured with a ketone body kit (Randox Laboratories Ltd., Crumlin, UK) and a free fatty acid quantification kit (BioVision, Mountain View, CA, USA), respectively.

### 4.4. Hematoxylin/Eosin (H&E) Staining

Hematoxylin/Eosin (H&E) staining was carried out using the method employed by Berezovskiĭ [30]. Liver and fat tissue samples were collected following euthanasia, fixed in 10% formalin buffered solution, and cut into 5-µm sections. Hematoxylin/eosin (H&E) staining was performed using standard techniques.

### 4.5. Hepatic and Fecal Lipid Analysis

Hepatic and fecal lipids were extracted according to the methods used by Tzang et al. [31], and concentrations of triglyceride and cholesterol were measured using a TG assay kit (Teco diagnostics, Anaheim, CA, USA) and a cholesterol commercial kit (Randox Laboratories Ltd., Crumlin, UK), respectively.

### 4.6. Hepatic Lipid Peroxidation Assay

TBARS were used as an index of the extent of lipid peroxidation, following the methods used by Buege and Aust [32].

### 4.7. TEAC Assay

Determination of TEAC was carried out using the method employed by Arnao et al. [33]. A dose-response curve was plotted for trolox, and antioxidant ability was expressed as the TEAC. The higher the TEAC value of a sample, the stronger the antioxidant activity.

### 4.8. Determination of Antioxidant Enzymes in the Liver

All antioxidant enzymes activity was determined after hepatic tissue was homogenized with phosphate-buffered saline solution at pH 7.0. GRd activity was determined according to the method used by Bellomo et al. [34]. GST activity was determined according to the method used by Habig et al. [35].

### 4.9. RNA Extraction and Real-Time RT-PCR

Real-time RT-PCR was performed to determine the level of gene expression in liver and adipose tissues. Total RNA was isolated using the TRIzol method (Life Technologies, Rockville, MD, USA) according to the manufacturer’s protocol. The cDNA was synthesized from the total RNA by reverse transcription PCR using a high-capacity cDNA reverse transcription kit (Applied Biosystems, Foster City, CA, USA) according to the manufacturer’s instructions. 

Real-time RT-PCR was conducted to evaluate gene expression levels using a Step One^TM^ RT PCR system (Applied Biosystems, Foster City, CA, USA). The reaction mixture (total volume 25 μL) contained 1× power SYBR green PCR master mix, 300 nM forward primer, 300 nM reverse primer, cDNA, and DEPC-H_2_O, as well as commercial reagents (Applied Biosystems, Foster City, CA, USA). The thermal profile was established according to the manufacturer’s protocol: 95 °C for 10 min for enzyme activation, followed by denaturing at 95 °C for 15 s, and annealing and elongation at 60 °C for 1 min, for a total of 40 cycles. The relative levels of gene expression were quantified using the ∆∆Ct method, which results in a ratio of target gene expression relative to equally expressed housekeeping genes (β-actin).

### 4.10. Statistical Analysis

The data were analyzed using an analysis of variance (ANOVA). A 0.05 probability level was used as the threshold for a statistically significant difference, and the differences between treatments were tested using the Least Significant Difference (LSD) test. All statistical analyses were performed using SAS (SAS Institute Inc., Hongkong, China, 2002).

## Figures and Tables

**Table 1:** Effect of deep sea water on the body weight, water intake, food intake, energy intake, organ weight, and adipose tissue weight in rats with obesity induced by a high-fat diet.

Obese Rat *	HFD Supplemented with DSW				
	Control	1×	2×	3×	5×
Initial Weight (g)	323.49 ± 2.35 ^a^	323.39 ± 2.36 ^a^	323.31 ± 2.30 ^a^	323.88 ± 1.79 ^a^	323.48 ± 1.97 ^a^
Final Weight (g)	510.35 ± 9.68 ^a^	478.46 ± 6.72 ^b^	480.23 ± 12.33 ^b^	459.61 ± 6.73 ^b^	457.95 ± 7.30 ^b^
Water Intake (mL/day)	44.32 ± 0.82 ^c^	48.76 ± 1.70 ^bc^	47.92 ± 2.42 ^bc^	59.49 ± 4.81 ^a^	53.93 ± 1.40 ^ab^
Food Intake (g/rat/day)	26.08 ± 0.89 ^a^	25.22 ± 0.74 ^a^	25.49 ± 0.69 ^a^	24.80 ± 0.36 ^a^	24.50 ± 0.76 ^a^
Energy Intake (kcal/rat/day)	129.59 ± 4.40 ^a^	125.27 ± 3.69 ^a^	126.62 ± 3.43 ^a^	123.22 ± 1.77 ^a^	121.70 ± 3.79 ^a^
Heart (mg/g rat)	2.72 ± 0.08 ^a^	2.83 ± 0.07 ^a^	2.79 ± 0.11 ^a^	2.91 ± 0.04 ^a^	2.79 ± 0.07 ^a^
Liver (mg/g rat)	27.19 ± 1.09 ^a^	25.86 ± 0.25 ^a^	24.09 ± 0.32 ^b^	24.00 ± 0.41 ^b^	23.91 ± 0.38 ^b^
Spleen (mg/g rat)	1.84 ± 0.06 ^a^	1.84 ± 0.06 ^a^	1.82 ± 0.07 ^a^	1.89 ± 0.08 ^a^	1.91 ± 0.12 ^a^
Lung (mg/g rat)	3.29 ± 0.12 ^a^	3.33 ± 0.14 ^a^	3.20 ± 0.09 ^a^	3.27 ± 0.08 ^a^	3.37 ± 0.09 ^a^
Kidney (mg/g rat)	6.22 ± 0.16 ^a^	6.09 ± 0.15 ^a^	6.28 ± 0.17 ^a^	6.20 ± 0.12 ^a^	6.17 ± 0.13 ^a^
Perirenal Fat (mg/g rat)	41.64 ± 2.87 ^a^	30.99 ± 1.55 ^bc^	34.00 ± 2.30 ^b^	24.42 ± 2.26 ^c^	28.04 ± 2.10 ^bc^
Epididymal Fat (mg/g rat)	26.09 ± 0.93 ^a^	23.34 ± 1.07 ^ab^	23.06 ± 1.90 ^abc^	20.57 ± 1.37 ^bc^	18.98 ± 1.43 ^c^

* The reported values are the mean ± SEM (*n* = 8). Mean values with different superscript letters in each row are significantly different (*p* < 0.05). HFD, high-fat diet; DSW, deep sea water.

**Table 2:** Effect of deep sea water on the serum biochemical indicators in rats with obesity induced by a high fat-diet.

Biochemical Indicators *	HFD Supplemented with DSW				
	Control	1×	2×	3×	5×
Glucose (mg/dL)	242.75 ± 11.73 ^a^	228.88 ± 9.82 ^ab^	205.38 ± 10.58 ^bc^	185.63 ± 14.68 ^cd^	168.38 ± 9.85 ^d^
Triglycerides (mg/dL)	116.63 ± 9.63 ^a^	79.13 ± 4.42 ^bc^	85.88 ± 4.99 ^b^	68.75 ± 4.43 ^bc^	67.25 ± 3.44 ^c^
Total Cholesterol (mg/dL)	60.54 ± 4.72 ^a^	47.00 ± 2.97 ^b^	46.86 ± 4.53 ^b^	52.73 ± 3.28 ^ab^	45.03 ± 2.78 ^b^
HDL-Cholesterol (mg/dL)	29.60 ± 1.39 ^a^	30.16 ± 2.12 ^a^	30.22 ± 1.55 ^a^	28.88 ± 1.38 ^ab^	24.48 ± 1.55 ^b^
LDL-Cholesterol (mg/dL)	8.51 ± 0.68 ^ab^	7.26 ± 0.30 ^b^	7.05 ± 0.34 ^b^	9.44 ± 0.75 ^a^	7.35 ± 0.58 ^b^
AST (U/L)	69.00 ± 2.95 ^a^	64.50 ± 3.26 ^ab^	61.38 ± 1.93 ^ab^	60.25 ± 2.62 ^ab^	57.00 ± 3.22 ^b^
ALT (U/L)	22.88 ± 1.36 ^a^	18.50 ± 1.28 ^b^	18.34 ± 0.75 ^b^	18.50 ± 1.25 ^b^	16.50 ± 0.71 ^b^
Uric Acid (mg/dL)	4.39 ± 0.20 ^a^	4.07 ± 0.25 ^a^	4.12 ± 0.32 ^a^	3.64 ± 0.28 ^a^	3.93 ± 0.14 ^a^
Creatinine (mg/dL)	0.76 ± 0.02 ^a^	0.79 ± 0.01 ^a^	0.75 ± 0.02 ^a^	0.75 ± 0.02 ^a^	0.74 ± 0.02 ^a^
Ketone Body (mmol/L)	1.94 ± 0.10 ^bc^	2.08 ± 0.14 ^bc^	1.76 ± 0.06 ^c^	1.84 ± 0.05 ^c^	2.25 ± 0.13 ^a^
Free Fatty Acid (mmol/dL)	0.99 ± 0.03 ^a^	0.79 ± 0.02 ^c^	0.93 ± 0.04 ^ab^	0.84 ± 0.05 ^bc^	0.74 ± 0.04 ^c^
Insulin (μg/L)	1.04 ± 0.12 ^a^	0.62 ± 0.05 ^b^	0.71 ± 0.07 ^b^	0.57 ± 0.07 ^b^	0.55 ± 0.02 ^b^
Mg^2+^ (mmol/L)	2.61 ± 0.10 ^b^	2.92 ± 0.08 ^ab^	3.00 ± 0.12 ^a^	2.97 ± 0.10 ^a^	3.01 ± 0.13 ^a^
Na^+^ (mmol/L)	165.63 ± 3.39 ^a^	162.50 ± 2.42 ^a^	166.50 ± 4.96 ^a^	164.50 ± 3.71 ^a^	160.13 ± 3.37 ^a^
K^+^ (mmol/L)	6.41 ± 0.26 ^b^	6.69 ± 0.18 ^ab^	6.73 ± 0.18 ^ab^	6.80 ± 0.28 ^ab^	7.18 ± 0.24 ^a^
Cl^−^ (mmol/L)	103.00 ± 1.88 ^a^	103.75 ± 1.41 ^a^	103.75 ± 2.31 ^a^	106.00 ± 2.56 ^a^	104.25 ± 1.58 ^a^
TEAC (nmol/mL)	242.75 ± 11.73 ^a^	228.88 ± 9.82 ^ab^	205.38 ± 10.58 ^bc^	185.63 ± 14.68 ^cd^	168.38 ± 9.85 ^d^

* The reported values are the mean ± SEM (*n* = 8). Mean values with different superscript letters in each row are significantly different (*p* < 0.05). AST, aspartate aminotransferase; ALT, alanine aminotransferase; DSW, deep sea water; HFD, high-fat diet; HDL, high-density lipoprotein; LDL, low-density lipoprotein; TEAC, trolox equivalent antioxidant capacity.

**Table 3:** Effect of deep sea water on hepatic antioxidant enzyme and total hepatic and fecal lipids, triglycerides, cholesterol, and magnesium in rats with obesity induced by a high-fat diet.

Obese Rat *	HFD Supplemented with DSW				
	Control	1×	2×	3×	5×
Hepatic					
TEAC (nmol/mg protein)	1.02 ± 0.01 ^a^	1.05 ± 0.01 ^a^	1.03 ± 0.04 ^a^	1.02 ± 0.01 ^a^	0.99 ± 0.03 ^a^
TBARS (nmol MDA eq./mg protein)	0.41 ± 0.08 ^a^	0.15 ± 0.04 ^b^	0.32 ± 0.06 ^ab^	0.12 ± 0.04 ^b^	0.37 ± 0.12 ^a^
GRd (nmol/mg protein)	9.76 ± 1.03 ^b^	11.17 ± 0.77 ^b^	9.89 ± 0.48 ^b^	14.34 ± 0.78 ^a^	16.30 ± 1.57 ^a^
GST (nmol/mg protein)	18.13 ± 1.48 ^ab^	14.84 ± 1.15 ^b^	17.39 ± 1.91 ^ab^	18.56 ± 1.82 ^ab^	21.25 ± 1.35 ^a^
Total Lipids (mg/g tissue)	66.63 ± 2.76 ^a^	63.93 ± 2.42 ^ab^	62.31 ± 3.04 ^ab^	52.11 ± 1.80 ^c^	56.92 ± 2.78 ^bc^
Triglycerides (mg/g tissue)	22.96 ± 1.82 ^a^	23.17 ± 1.44 ^a^	17.24 ± 1.85 ^b^	17.72 ± 1.82 ^b^	17.91 ± 0.83 ^b^
Cholesterol (mg/g tissue)	9.55 ± 0.98 ^a^	7.98 ± 0.66 ^ab^	7.47 ± 1.05 ^ab^	6.50 ± 0.68 ^b^	6.77 ± 0.85 ^b^
Magnesium (mg/g tissue)	57.40 ± 7.95 ^b^	74.69 ± 8.19 ^ab^	66.92 ± 6.97 ^ab^	75.42 ± 5.78 ^ab^	85.81 ± 5.49 ^a^
Fecal					
Total Lipids (mg/g dried fecal)	51.52 ± 1.69 ^b^	61.46 ± 5.67 ^a^	64.04 ± 2.29 ^a^	68.22 ± 1.50 ^a^	63.62 ± 3.08 ^a^
Triglycerides (mg/g dried fecal)	7.93 ± 0.30 ^b^	8.70 ± 0.72 ^b^	12.34 ± 1.00 ^a^	11.60 ± 0.54 ^a^	13.09 ± 1.23 ^a^
Cholesterol (mg/g dried fecal)	6.70 ± 0.29 ^b^	6.48 ± 0.31 ^b^	8.30 ± 0.27 ^a^	7.95 ± 0.47 ^a^	8.33 ± 0.41 ^a^

* The reported values are the mean ± SEM (*n* = 8). Mean values with different superscript letters in each row are significantly different (*p* < 0.05). DSW, deep sea water; HFD, high-fat diet; TBARS, thiobarbituric acid reactive substances; GRd, glutathione reductase; GST, glutathione S-transferase; TEAC, trolox equivalent antioxidant capacity.

## Abbreviations

ACO	acyl-CoA oxidase
ALT	alanine transaminase
AMPK	AMP-activated protein kinase
AST	aspartate aminotransferase
ATGL	adipose triglyceride lipase
CPT	carnitine palmitoyl transferase
GRd	glutathione reductase
GST	glutathione S-transferase
HDL cholesterol	high-density lipoprotein cholesterol
HSL	hormone-sensitive lipase
IL	interleukin
LDL cholesterol	low-density lipoprotein cholesterol
MDA	malondialdehyde
PAI	plasminogen activator inhibitor
PPAR	peroxisome proliferator-activated receptor
SREBP	sterol regulatory element binding protein
TBARS	thiobarbituric acid reactive substances
TEAC	Trolox equivalent antioxidant capacity
TNF	tumor necrosis factor
